# Sad mood and poor sleep are related to task-unrelated thoughts and experience of diminished cognitive control

**DOI:** 10.1038/s41598-020-65739-x

**Published:** 2020-06-02

**Authors:** David Marcusson-Clavertz, Oscar N. E. Kjell, Jinhyuk Kim, Stefan D. Persson, Etzel Cardeña

**Affiliations:** 1 Department of Psychology, Lund University, Lund, Sweden; 2 Department of Psychology, Ludwig Maximilian University of Munich, Munich, Germany; 3 Department of Informatics, Shizuoka University, Shizuoka, Japan

**Keywords:** Human behaviour, Health care

## Abstract

Previous studies have indicated that a sad mood and sleep deprivation increase mind wandering, but it is unclear whether these associations reflect reduced effort in concentrating on the task at hand or diminished cognitive control. In an internet-based experiment, participants completed a sleep disturbance questionnaire followed by a complex span task and a 2-back task with thought-sampling probes. Subsequently, participants underwent a positive, neutral, or negative mood induction prior to repeating the 2-back. The results (*N* = 504) replicated the finding of increased task-unrelated thoughts following sad mood induction, *B* = 0.56 (*SE* = 0.14), *p* < 0.01, *d* = 0.31. Unguided thoughts were increased following sad mood induction, *B* = 0.31 (0.13), *p* = 0.02, but working memory did not significantly moderate this association (*p* = 0.31). People reported a lower degree of trying to concentrate on the 2-back after the sad mood induction, *B* = −0.07 (0.04), *p* = 0.04, but actual performance was not affected (*p* = 0.46). Sleep disturbances showed small associations with task-unrelated, *B* = 0.23 (0.08), *p* < 0.01, and unguided thoughts, *B* = 0.32 (0.08), *p* < 0.01. This study strengthens the evidence that a sad mood and poor sleep relate to mind wandering.

## Introduction

Across virtually all everyday activities, people frequently think about task-unrelated matters (mind wandering)^1^, a state in which mentation is decoupled from the external environment^2^. Mind wandering is more likely to occur during a sad mood^3^ and after sleep deprivation^4^. Individuals with greater cognitive resources mind wander less during cognitive tests, supporting the view that at least some types of mind wandering relate to cognitive failures^5^. Applied research has related mind wandering to diminished performance on everyday tasks like reading^6^ and driving^7^, suggesting that it is imperative to understand the ebb and flow of mind wandering to minimize its costs while maximizing its potential benefits (e.g., creativity, planning)^8^. However, researchers vary greatly in how they define mind wandering or which of its aspects they have evaluated, which makes it difficult to synthesize the literature^9^.

To add clarity, Seli and colleagues applied the family-resemblance framework and defined mind wandering as having no necessary features, but all exemplars of mind wandering should share at least some common features (e.g., task-unrelated, stimulus-independent, unguided, guided, and meandering thoughts or images)^9^. They recommended researchers clarify which feature of mind wandering they are measuring, and, if feasible, examine multiple features to understand their relations and distinctiveness. For instance, task-unrelated thoughts that are unguided (i.e., spontaneous, uncontrolled) versus those that are guided (i.e., intentional, controlled) relate differently to personality and clinical variables^10^ and task contexts^11^. In this online experiment, we examined three properties of mental activity pertaining to mind wandering: 1) task-unrelated thoughts, 2) unguided thoughts, and 3) the degree people tried to concentrate on the task. We also evaluated how these properties relate to mood, working memory capacity, and sleep disturbances. In our review of the relevant mind wandering literature, most studies measured mind wandering in the context of an explicit task and so we use the term “task-unrelated thoughts” interchangeably with “mind wandering” in this context.

Most relevant to the present study are two reports of between-subjects experiments on mood and mind wandering with small sample sizes (*N* ≤ 20 per condition). One of the studies, measuring task-unrelated thoughts with a post-task-questionnaire, observed higher amounts of task-unrelated thoughts following sad mood induction compared to neutral and happy (a medium-to-large omnibus effect, *η*^2^ = 0.12)^3^. The other study, measuring task-unrelated thoughts by prompting participants to list all their thoughts at the end of the task, showed increased task-unrelated thoughts following both happy and sad mood inductions^12^. Experience sampling studies in ecological settings show associations between momentary negative affect and task-unrelated thoughts in daily life^1,13^. However, it is not clear whether a sad mood increases task-unrelated thoughts by reducing effort or controllability of thoughts (e.g., by increasing cognitive load or interfering with inhibitive cognitive processes), or through other means.

One view of mood states is that they influence motivation by prioritizing self-relevant processing^3,14^ at the expense of task performance, and by biasing attention towards emotionally salient information^15^. Participants who undergo mood induction procedures may therefore give less priority to the neutral monotonous tasks they are asked to complete in favour of emotionally salient self-processing. Another view is that mood states modulate executive cognitive control functioning^16,17^, which might result in greater difficulty preventing task-unrelated thoughts, specifically unguided (i.e., spontaneous) thoughts^18^. Although the results pertaining to happy mood and mind wandering are mixed^3,12^, it may be conjectured that happy mood will induce more unguided, exploratory thoughts, according to the broaden-and-build theory of positive emotions^19^. On the other hand, if happy mood increases motivation or commitment to current tasks, it is possible that it instead increases the degree people are trying to concentrate on the task at hand and reduces task-unrelated thoughts.

In addition to mood, mind wandering may be related to higher-order cognitive abilities, including working memory. Working memory can be defined as a multicomponent system that temporarily maintains and manipulates information in the mind during processing or distraction^20^. It includes domain-specific components involved with storage of particular information and a domain-general executive component that regulates the flow of information in and out of awareness and across the domain-specific systems^21,22^. McVay and Kane postulated that the executive component prevents task-unrelated thoughts^18^. Support for this account was provided by a meta-analysis of 20 studies finding a very small but statistically significant negative correlation between executive cognitive resources and task-unrelated thoughts (*r* = −0.11)^23^. The relation between task-unrelated thoughts and working memory capacity appears to be stronger during more difficult tasks, as one study showed a moderately large and significant correlation during a high-demanding condition of a memory task (*r* = −0.32), but not in a low-demanding memory task (*r* = −0.01)^5^. An experience sampling study indicated that those with greater working memory capacity restrained their daily life task-unrelated thoughts more effectively at times when they were trying to concentrate^24^. Another experience sampling study indicated that working memory capacity moderated the relation between having a negative daydreaming style and frequency of daily task-unrelated thoughts^25^. Our interpretation of these findings is that individuals with greater working memory capacity have more flexible executive cognitive ability and can adjust the flow of information so that they minimize task-unrelated thoughts based on their willingness to concentrate on the task or the likelihood that the mind wandering content will disturb them. If a sad mood reduces the willingness to concentrate on a neutral task or interfere with the cognitive capacity to focus (e.g., by reducing controllability of thoughts), then individuals with superior working memory capacity may be better able to regulate the effects of moods and thus have fewer unguided thoughts.

Another candidate for disrupting the ability to prevent mind wandering is poor sleep. Surprisingly few studies have examined the relation between sleep and mind wandering despite some relatively large associations reported. A study showed that one night of sleep deprivation significantly increased task-unrelated thoughts in the laboratory, but only for the more demanding of two task conditions (a large effect, Cohen’s *d* = 0.98)^4^. A survey study found medium-to-large negative and significant correlations between questionnaires of global sleep quality and task-unrelated thoughts or daydreaming frequency across a number of studies (*r*s ≈ 0.30–40)^26^. Little is known about why poor sleep predicts mind wandering, but as sleep is related to working memory ability^27^, one possibility is that sleep disruptions lead to cognitive difficulties in preventing unguided thoughts (i.e., thoughts may become less controlled after poor sleep).

Our aim was to contribute to the mind wandering and mood literature by examining multiple mind wandering-related features, namely task-unrelated thoughts, unguided thoughts, and the degree people tried to concentrate on the task. We were also interested in testing which predictor, sleep, mood, and/or working memory capacity would uniquely predict each of these mind wandering-related features when included in one large model. As reports of mind wandering are weakly related to measures of social desirability tendency^10^, we also included the latter as a covariate to reduce the risk that potential findings might be due to self-report biases. We did not have a hypothesis about the effects of happy mood induction on the mind-wandering related features as previous research have yielded mixed results on the effect on task-unrelated thoughts. Our hypotheses were as follows:

1. Compared to a neutral mood, a sad mood will increase: a) task-unrelated thoughts and b) unguided (i.e., spontaneous) thoughts, and reduce c) performance on working memory test and d) efforts to concentrate on the task.
2. The association between working memory and unguided thoughts will be more negative in the sad mood condition than in the neutral mood condition.
3. Sleep disturbances will be related to more: a) task-unrelated thoughts and b) unguided thoughts.

## Methods

### Participants

We pre-registered (https://aspredicted.org/tv2uf.pdf) that we would collect data from 600 individuals. As 601 completed the data collection through the recruitment site Academic Prolific^28^, we dropped one person who did not follow the instructions for the Operation span (OSPAN) task. Out of the 600 individuals, we excluded 26 individuals who failed to answer the two control items correctly. The remaining sample included 241 men and 330 women, and 3 who reported “other”. The mean age was 25.87 (*SD* = 4.99) and mean score on the 1–9 socioeconomic ladder item was 5.34 (*SD* = 1.56). Participants indicated that their nationality was United Kingdom (*n* = 216), United States (*n* = 74), Poland (*n* = 66), Portugal (*n* = 40), Italy (*n* = 24), and Germany (*n* = 23), among other countries.

### Materials

#### Patient Health Questionnaire-2 (PHQ-2)

This self-report instrument includes two items about depression. A sample item is “Over the last 2 weeks….Little interest or pleasure in doing things” answered on a 4-point scale with options 0 (*Not at all*), 1 (*Several days*), 2 (*More than half the days*) and 3 (*Nearly every day*). A study provided support for its reliability and convergent validity with professional health interviews^29^. This instrument is integrated into the Academic Prolific platform as a pre-screener and we invited participants to complete this study if they scored at most 1 on each of the two items.

#### Control items

Two control items were used to examine whether participants were paying attention to the online experiment. The first control item was presented last amongst the items in the PROMIS Sleep Disturbance Short Form questionnaire, and it asked respondents to “Please select alternative ‘2 Poor’ here.” The second item was presented during the practice round of the Visuospatial 2-back task, and it stated, “On this question please select alternative ‘4 Quite a bit’.” Participants that failed to answer these two items correctly were removed from the analyses. This kind of attention check procedure has been found to increase the statistical power of a dataset^30^.

#### PROMIS sleep disturbance short form

This self-report instrument measures sleep disturbances in the last week^31^. It includes 8 items about sleep quality. A sample item is “In the past SEVEN (7) DAYS…. I had difficulty falling asleep” answered on a 5-point scale with options 0 (*Not at all*)*, 1* (*A little bit*)*, 2* (*Somewhat*)*, 3* (*Quite a bit*), and 4 (*Very much*). A study provided support for its validity and reliability and showed that the short form provides better measurement precision than standard sleep quality questionnaires despite having fewer items^31^.

#### Social Desirability Scale (SDS)

This self-report instrument measures the tendency to produce socially desirable responses^32^. The revised version excludes an item on drug use and consists of 16 items. Participants are asked to indicate if each statement describes them by answering “True” or “False”. A sample item is “In conversations I always listen attentively and let others finish their sentences,” where “True” would be the socially desirable response.

#### Operation Span Task (OSPAN; Klingon Span)

We used an adapted OSPAN task to measure individual differences in working memory capacity. Our version of the test required participants to recall Klingon letters (a fictional alphabet used in Star Trek movies) while also solving simple mathematical equations. We programmed the task in JsPsych (version 6.0.3)^33,34^ and adapted it to be similar to the Klingon span task from previous research^35^. Hicks and colleagues reported that using Klingon letters, which are more difficult to rehearse than ordinary letters, resulted in better concurrent validity (i.e., greater association with measures of general intelligence)^35^.

Participants first completed a practice block of the primary task, the Klingon memory part of the test. Letters were shown sequentially for 1200 ms and at the end of the trial participants were shown the entire pool of 9 letters plus a blank space and were asked to respond which letters were shown in which position order by typing the corresponding number next to the letter.

Participants next completed a practice block of the secondary task prompting them to solve 15 mathematical equations. In each trial a simple equation was presented with a question mark at the end. After having mentally solved the problem participants pressed spacebar. The question mark was then replaced with a proposed solution and participants were required to indicate whether the solution was true or false by pressing the left or the right arrow within 3000 ms. We gave trial-by-trial feedback (“correct” or “incorrect”) on the response to every mathematical equation to encourage participants to comply. The feedback was shown for 1000 ms in the practice and 250 ms in the experimental trials.

Participants then completed a final practice block that included both tasks (two trials each with two letters and two mathematical equations). At the end of each trial participants were asked to recall the letters and their positions. At the end of the practice, participants were shown their mean response time and accuracy on the mathematical equations.

**Figure 1:**
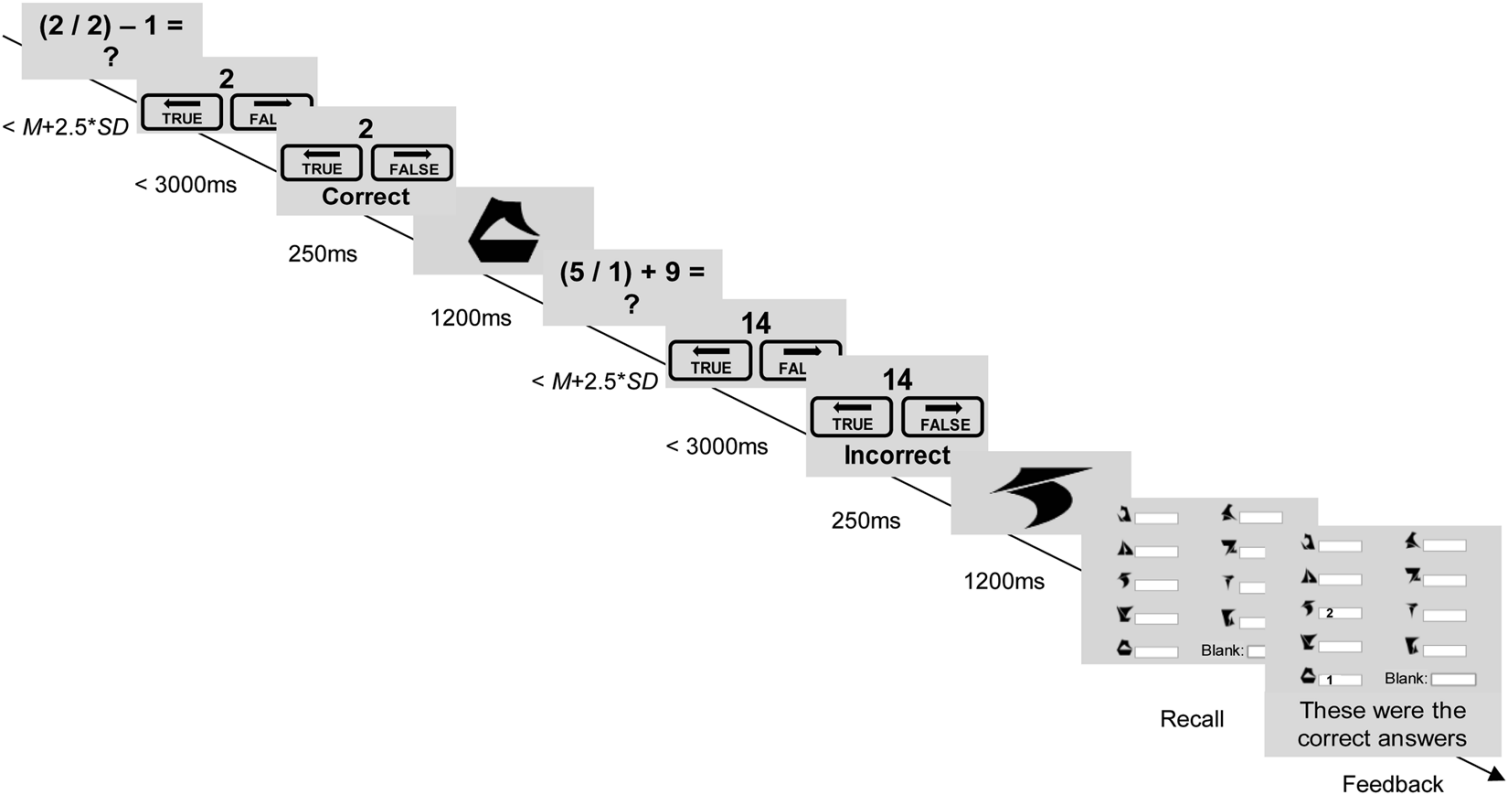
An overview of the Operation span task with Klingon letters. In each trial participants indicated whether a proposed mathematical solution was true or false, followed by brief feedback (“correct” vs. “incorrect” for 250 ms). Then, a Klingon letter was shown for 1200 ms which they were asked to remember. After two to five letters they were prompted to recall the letters and their position order.

In the experimental blocks, participants completed 12 trials with two, three, four, or five letters each. Thus, total OSPAN outcome ranged from 0 to 42 letters recalled in correct position order. Participants were instructed to obtain at least 85% accuracy on the secondary task (the equations). In addition to the trial-by-trial feedback on the secondary task, the sixth and ninth trials were followed by feedback on the cumulative accuracy rates. Trials in which participant took longer than *M* + 2.5 *SD* (person-specific, based on the second practice round) were counted as errors. We used the recommended partial-credit score computation^36^. Figure 1 shows an overview of the task.

#### Visuospatial 2-Back with Thought-Sampling Probes

This task includes a 3 × 3 matrix with 8 blank squares and 1 red square in each trial. Participants were required to indicate whether the location of the red square matched the location two trials earlier (i.e., 2-back match trials). In each trial, each stimulus was shown for 1500 ms followed by a 1000 ms inter-stimulus interval (a blank matrix). Participants had to press *M* for match trials (20%) and *C* for control trials (80%) using the keyboard. Response interval was 2500 ms. There were 190 trials divided in 10 blocks with 10, 20, or 30 trials each. We created two semi-random trial sequences that were used for pre vs. post-induction conditions (counterbalanced across participants).

We computed a *d’* performance score by subtracting the false alarm rate (i.e., proportion of control trials incorrectly answered) from the hit rate (i.e., proportion of match trials correctly answered). This computation mitigates the impact of response biases (e.g., excessive tendency to indicate a match). We applied the probit transformation to both hit rates and false alarm rates following recommendations from previous research^37^. We computed the *d*’ score for each block separately. To avoid using the probit function on 0 s and 1 s, we added one match trial and four control trials with scores of 0.5 for every block (as there was a 4:1 ratio of control trials).

At the end of each of the 10 blocks, participants were prompted by a “STOP” screen to recall their most recent thought followed by the following three questions:

1. Right before you saw the word “STOP”, **what were you thinking about**?
  I was thinking about the task
  I was thinking about something else
2. Right before you saw the word “STOP”, were you **in control of** (**guiding)** your thoughts?
  Yes, I controlled (guided) the thoughts
  No, the thoughts came to me spontaneously
3. Right before you saw the word “STOP”, how much were you **trying to concentrate** on the task?

0 Not at all

1 Slightly

2 Somewhat

3 Quite a bit

4 Extremely

**Figure 2:**
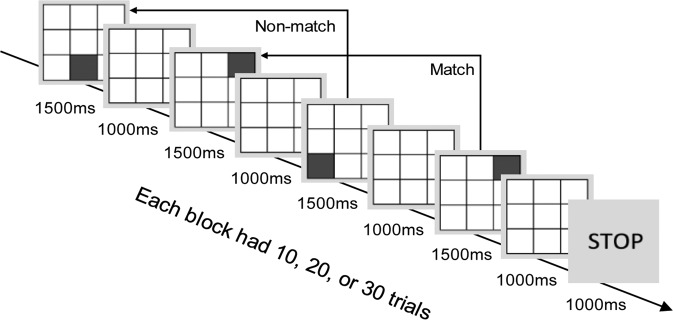
An overview of the 2-back task with thought-sampling probes. In each trial, a filled square is shown in a 3 × 3 matrix. Participants were asked to indicate whether the filled square is located in the same position as it was two trials earlier. At the end of each block, a STOP probe prompted participants to answer three questions about their mental activity right before the probe: whether they were thinking about the task, whether they were in control of (guiding) their thoughts, and how much they tried to concentrate on the task.

In support of these thought probe measures, reports of *task-unrelated thoughts* have been triangulated against neurocognitive and behavioural measures^2^. One study indicated that reports of *unguided thoughts* (i.e., the sense of not controlling one’s thoughts) loaded negatively on a component termed control/awareness (e.g., high awareness of mentation, narrow awareness span, clear thoughts, easy to remember thoughts) that was distinct from the mind wandering component^38^. Reports of *trying to concentrate* have been shown to relate to behavioural measures of attention-restraint ability and working memory capacity as well as reports of fewer task-unrelated thoughts^24^. Figure 2 shows an overview of the task procedure.

#### Mood induction procedures

We used the same 8-min mood induction procedures as reported in a previous study^39^. One group underwent a *happy* mood induction procedure comprising a 4 min video clip, Hakuna Matata (from the animated motion picture *The Lion King)*, followed by 4 min of the instrumental piece of the *Prélude et Mazurka* (from the ballet “Coppélia, Act 1” based on music by Léo Delibes). Another group underwent a *neutral* mood induction including a 4 min documentary clip about Magnets (edited from the series *Modern Marvels*) followed by 4 min of the instrumental piece *Variations for Winds, Strings and Keyboards* (by Steve Reich). A third group underwent a *sad* mood induction including a 4 min video clip showing the death of Mufasa (from the animated film *The Lion King)* followed by 4 min of the *Adagio for Strings, Op. 11* (by Samuel Barber). While listening to the music, all groups were asked to close their eyes. The previous study showed that compared to the neutral mood induction, happy mood induction greatly increased self-reported joviality whereas sad mood induction greatly increased self-reported sadness^39,40^.

#### Self-Assessment Manikin (SAM)

As a manipulation check of the mood induction procedures, we administered a single mood item. The instructions were: “The following question is about your current mood. The scale consists of a number of images that represent negative to positive mood. Indicate to what extent you feel this way **right now**, that is, at the present moment.” And participants answered on a nine-point scale from 1 (*very unhappy*) to 9 (*very happy*) with SAM images to represent each mood state.

### Procedure

Volunteers who passed the PHQ-2 screening criteria could see the advert on Prolific (https://prolific.ac/). They were informed about the purpose of the study, including the use of sad, happy, and neutral mood induction procedures. All participants gave informed written consent and were then asked to complete the PROMIS questionnaire, the SDS, and the OSPAN in that order. They were next asked to complete the visuospatial 2-back with 10 thought-sampling probes followed by the SAM item on mood. As we intended these measures to measure individual differences at baseline, we presented them prior to the mood induction.

Participants were then divided into three groups who underwent either the happy, neutral, or sad mood induction procedure. To reduce the risk of demand characteristics we asked them to give honest self-reports and instructed them not to worry if they did not immediately feel the effect of the mood induction, that we were testing different procedures, that no procedure would work on everyone, and that they should just try their best to feel the targeted mood. After the mood induction, all groups were asked to complete the SAM item on mood and the 2-back with 10 thought sampling probes again. Participants who underwent neutral or sad mood induction procedures were asked to undergo the happy mood induction procedure to increase the chances that they completed the study in a happy mood. At the end, participants were compensated with £5.6 each. The study was approved by the regional ethics review board at Lund University (regionala etikprövningsnämnden, Lund; 2017/308) and the methods were performed in accordance with the European Code of Conduct for Research Integrity (All European Academics, ALLEA).

### Analyses

We used an α of 0.05 (two-tailed) to test our hypotheses, and a slightly more conservative 0.01 (two-tailed) for the exploratory analyses to reduce the risk of false positives. As pre-registered, we performed multilevel analyses with unstructured covariance structure and maximum likelihood estimation technique for continuous outcomes. We did not specify estimation technique for the binomial outcomes but used the logit link transformation and the Laplace estimation, which approximates maximum likelihood; we have used this estimation in previous research^25^. Our default choice was to model a random intercept, and the significant variance in intercepts across all models supported this choice.

We treated the probe outcome *trying to concentrate* as continuous (analysed with PROC MIXED in SAS). The variable was skewed with few extremely low scores but analysing it as an ordinal variable using PROC GLIMMIX in SAS yielded similar outcomes. We report the pre-registered mixed model with the continuous raw variable. For the analyses on binomial outcomes, *B* coefficients represent log odds ratios. We transformed them to odds ratios and Cohen’s *d* to simplify interpretations.

## Results

### Screening

In addition to the 26 excluded people who failed the control items (4% of the total sample of 600 individuals), we also pre-registered screening criteria for the OSPAN and 2-back. There were 60 participants (10% of the total sample) who failed to comply with the OSPAN task instructions of having at least 85% accuracy on the secondary task. We treated their OSPAN scores as missing. There were 17 people (3% of the total sample) who failed to obtain a positive *d*’ score on the 2-back prior to induction and we treated their 2-back performance and thought probe data as missing. In total, the multilevel analyses were based on 504 individuals (84% of the total sample) and 10,080 observations. There was no significant association between inclusion status and mood group, Χ^2^(2) = 0.18, *p* = 0.913.

### Descriptive summary of research measures

The descriptive stats are reported in Table 1. Participants recalled about 22 of the 42 Klingon letters in the OSPAN task. Their average sleep disturbances were close to midpoint of the 0–4 PROMIS scale. As for the SDS, participants chose the socially desirable response on about 58% of the items. Across the 20 probes during the 2-back task, participants on average reported having task-unrelated thoughts 21% of the time and unguided thoughts 30% of the time. The average score for “trying to concentrate” was 2.98 on the 0–4 scale in which 3 was labelled “quite a bit.” All measures showed high reliability (α above 0.80) except for the SDS which had barely acceptable reliability (α = 0.66).

**Table 1:** Descriptive statistics for research measures.

Measures	Score	*N*	*M*	*SD*	Skew	Kurtosis	Cronbach’s α
OSPAN Score	Sum	514	22.49	9.71	0.04	−0.93	0.83
PROMIS Sleep Disturbances	Mean	574	1.65	0.83	0.32	−0.62	0.90
Social Desirability Tendency	Rate	574	0.58	0.19	−0.32	−0.10	0.66
2-back Task-unrelated thoughts	Rate	557	0.21	0.21	1.14	1.16	0.84
2-back Unguided thoughts	Rate	557	0.30	0.26	0.71	−0.34	0.88
2-back Trying to concentrate	Mean	557	2.98	0.71	−0.56	−0.06	0.95
2-back *d*’ score	Mean	557	1.61	0.56	−1.16	0.78	0.94

Note. We estimated reliability for each 2-back variable by computing the score for each block type (i.e., 10 vs. 20. vs 30 trials per block) and then treating those three block types as scale items.

### Mood manipulation check

In brief, the effectiveness of the mood induction procedures was supported. There were some missing scores on the SAM items so *n*s were 178, 190, and 166 for sad, happy, and neutral groups, respectively. Sad mood induction reduced people’s mood ratings from *M*_pre_ = 4.66 (*SD* = 1.51) to *M*_post_ = 2.66 (*SD* = 1.29), Hedges *G* = −1.41, *p* < 0.001. Happy mood induction increased people’s mood ratings from *M*_pre_ = 4.56 (*SD* = 1.49) to *M*_post_ = 5.95 (*SD* = 1.23), Hedges *G* = 1.01, *p* < 0.001, whereas the neutral mood induction showed a small nonsignificant increase in mood ratings, *M*_pre_ = 4.81 (*SD* = 1.33) to *M*_post_ = 5.02 (*SD* = 1.29), Hedges *G* = 0.16, *p* = 0.050.

Critically, compared to neutral mood induction, happy mood induction showed a large increase in mood, Hedges *G* = 0.91, *p* < 0.001, whereas sad mood induction showed a large reduction, Hedges *G* = −1.42, *p* < 0.001. Because the mood groups did not differ in age or gender (*p*s > 0.5), age and gender were not analyzed further. We will next summarize the responses to the thought sampling probes across mood inductions before reporting the multilevel analyses.

### Descriptive summary of 2-back outcomes across mood conditions

**Figure 3:**
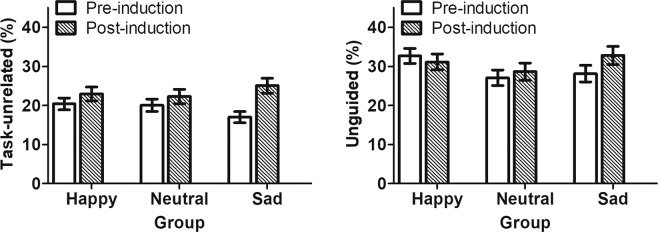
Descriptive summaries of the percentage of times participants reported task-unrelated and unguided thoughts in response to the 2-back probes. The bars show raw scores across individuals from happy (*n* = 199), neutral (*n* = 174), and sad (*n* = 184) groups. The error bars reflect standard errors of the means.

**Figure 4:**
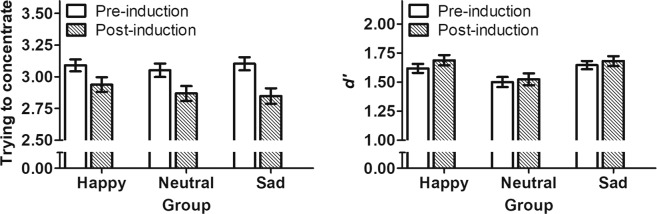
Descriptive summaries of the extent participants reported trying to concentrate on the 2-back test and their *d*´ performance scores. The bars show raw scores across individuals from happy (*n* = 199), neutral (*n* = 174), and sad (*n* = 184) groups. The error bars reflect standard errors of the means.

Figure 3 shows the descriptive summaries for the binomial outcomes, whereas Fig. 4 summarizes the continuous outcomes. Notably, those who underwent sad mood induction reported an 8%-unit increase in task-unrelated thoughts following induction, whereas those who underwent neutral mood induction reported a 2%-unit increase (Fig. 3). We next performed multilevel analyses for each of these four outcomes to test our hypotheses.

### Hypothesis testing with multilevel analyses

#### Task-unrelated thoughts

Table 2 shows the multilevel analysis with task-unrelated thoughts as the outcome. Compared to neutral mood induction procedure, sad mood induction increased task-unrelated thoughts, supporting our hypothesis. Specifically, sad mood induction increased the odds of reporting task-unrelated thoughts with 75% compared to neutral induction, which corresponds to a small-to-medium effect (Cohen’s *d* = 0.31). The hypothesis that sleep disturbances would relate to task-unrelated thoughts was also supported (see Table 2). That is, the odds of reporting task-unrelated thoughts were 26% higher in individuals with 1 *SD* above grand average in sleep disturbances. This odds ratio corresponds to Cohen’s *d* = 0.13 (i.e., a small association).

**Table 2:** Multilevel results with probe-reported task-unrelated thoughts, unguided thoughts, trying to concentrate on the task, and 2-back *d*’ score as outcomes (*N* = 504).

Predictor	Outcome							
	Task-unrelated thoughts		Unguided thoughts		Trying to concentrate		2-back *d*’ score	
	*B (SE)*	*p*	*B (SE)*	*p*	B (SE)	*P*	*B (SE)*	*p*
Intercept	−1.87 (0.14)	<0.001***	-1.48 (0.15)	<0.001***	3.07 (0.06)	<0.001***	1.55 (0.04)	<0.001***
Social Desirability Scale (*z*)	−0.07 (0.07)	0.326	0.08 (0.08)	0.342	0.02 (0.03)	0.450	−0.03 (0.02)	0.256
Operation Span (OSPAN, *z*)	−0.00 (0.08)	0.967	−0.07 (0.09)	0.416	0.03 (0.03)	0.297	0.18 (0.02)	<0.001***
PROMIS Sleep Disturbances (*z*)	0.23 (0.08)	0.002**	0.32 (0.08)	<0.001***	−0.06 (0.03)	0.046*	−0.06 (0.02)	0.010**
Induction	0.13 (0.10)	0.201	0.04 (0.09)	0.635	−0.19 (0.03)	<0.001***	0.03 (0.02)	0.179
Happy group	−0.06 (0.19)	0.744	0.35 (0.21)	0.091	0.04 (0.08)	0.651	0.12 (0.06)	0.036*
Sad group	−0.27 (0.19)	0.162	0.06 (0.21)	0.774	0.05 (0.08)	0.531	0.11 (0.06)	0.044*
Induction × Happy	0.07 (0.14)	0.622	−0.12 (0.13)	0.344	0.05 (0.03)	0.177	0.05 (0.03)	0.097
Induction × Sad	0.56 (0.14)	<0.001***	0.31 (0.13)	0.020*	−0.07 (0.04)	0.037*	0.02 (0.03)	0.462
OSPAN (z) × Induction × Happy	0.08 (0.09)	0.366	0.13 (0.09)	0.132	-0.03 (0.02)	0.194	0.01 (0.02)	0.692
OSPAN (z) × Induction × Sad	−0.02 (0.09)	0.835	0.09 (0.09)	0.310	0.06 (0.02)	0.014*	-0.02 (0.02)	0.299

***p < 0.001, **p < 0.01, *p < 0.05.

#### Unguided thoughts

The second multilevel analysis indicated that sad mood induction increased unguided thoughts, consistent with our expectations (see Table 2). That is, sad mood induction increased the odds of reporting unguided thoughts with 36%, corresponding to a small effect, Cohen’s *d* = 0.17. We did not find support for the hypothesis of OSPAN performance moderating the effect of sad mood induction on unguided thoughts. As Table 2 shows, there was support for the hypothesis that sleep disturbances relate to unguided thoughts: the odds of reporting unguided thoughts were 38% higher in those individuals with 1 *SD* above grand average in sleep disturbances, corresponding to a small association, Cohen’s *d* = 0.18.

#### Trying to concentrate on the task

The third multilevel analysis showed that people generally tried less to concentrate on the task the second time they performed it. Specifically, the degree people tried to concentrate was reduced by 0.19 units following neutral mood induction (see Table 2). In support of our hypothesis, sad mood induction weakly reduced how much people tried to concentrate on the task: compared to neutral induction, sad induction reduced the average score on the 5-point scale with 0.07 units.

#### Performance (d’ score)

As Table 2 shows, the fourth multilevel analysis did not provide support for the expectation that a sad mood would reduce *d*’. However, the neutral mood group showed lower performance than the other two groups prior to the induction (*p*s = 0.04, see Fig. 4), indicating that the randomization was not optimal for this analysis. As for the baseline measures, OSPAN performance and sleep disturbances reports were each associated with *d*’. To summarize the results on social desirability, it did not relate to any of the four outcomes in the multilevel analyses.

We re-ran all four multilevel analyses with the full sample (*N* = 600) without any of the screening filters. All five significant and nonsignificant findings pertaining to our hypotheses remained the same.

## Discussion

Using 8-min mood induction procedures administered online, we obtained a small-to-medium effect of sad mood induction on task-unrelated thoughts, replicating previous laboratory research^3^ and contributing with a larger *N* and pre-registered analyses. We extended this effect of sad mood induction to increased unguided (spontaneous) thoughts and reduced tendency to concentrate on the task at hand, although these associations were very small. We also replicated the association between perceived sleep disturbances and task-unrelated thoughts, consistent with laboratory^4^ and survey research^26^, although the association was small in our study.

As the platform for internet-based behavioural experiments grows^28^, this study offers *proof of concept* for administering mood induction procedures online and reliably measuring individual differences in behavioural performance and probe-caught self-reports during complex cognitive tasks. Internet-based data collection facilitates conducting high-power studies with quick data collection and can contribute to the investigation of reproducibility of psychological research and complement laboratory research. In addition, we found support for the hypothesis that sleep disturbances relate to the tendency to have unguided thoughts. We did not find support for an effect of sad mood induction on actual performance on the 2-back task, nor did we find a moderating role of working memory capacity (measured with the OSPAN task) on the effect of sad mood induction on unguided thoughts. Another recent study, which tested happy vs. sad mood induction, also failed to find a moderating role of working memory capacity on mood and mind wandering during a reading and a fast go/no-go task^41^. As working memory capacity may play a different role in the cultivation of mind wandering in less challenging tasks^5^, such as 1-back or breath-counting tasks, we cannot generalize our results to those contexts.

As we only found weak effects of sad mood induction on effort and the sense of guiding one’s thoughts, it seems unlikely they can explain the upsurge in task-unrelated thoughts. Instead, it may be more informative to examine emotion regulation strategies as a moderator of the effect of sadness on task-unrelated thoughts. Some people may prioritize self-processing during a sad mood at the expense of externally directed attention^42^, others may instead try harder to concentrate on actual task at hand as a means to avoid sad thoughts (e.g., carrying out simple chores to cool down the temper while being upset). In other words, sad mood may reduce effort to concentrate on the task in some people and increase it in others depending on the emotion-regulation strategies. Likewise, poor sleep may prime task-unrelated thoughts at the expense of external attention depending on mood regulation strategies (e.g., approach vs. avoidance strategies).

Variability in emotion regulation strategies may also explain the failure to find any moderation of working memory capacity on task-unrelated and unguided thoughts. Another possibility is that greater mood changes or greater time-on-task^23^ are needed to produce a change related to working memory capacity than those observed in our study. Participants in this study reported on average spending quite a bit of effort on the task, whereas only a few reported not trying to concentrate at all. With greater time-on-task there would likely be more variability both within- and between-person. Thus, we should be cautious about generalizing our findings to contexts with stronger mood states, greater time-on-task, and differing motivation. We also acknowledge that we measured mind wandering during a specific cognitive task performed at the computer and the ecological validity of similar laboratory-based measures is limited (e.g., such measures being less meaningful and yielding lower commitment)^24^. In defence of our approach, we did replicate experience-sampling findings on daily life mind wandering with mood^1,13^ and survey research on sleep^26^.

We extended the previous literature on sleep disturbances and mind wandering by showing that the former relates to two features of mind wandering: task-unrelatedness and sense of not guiding’s one’s thoughts. These associations were small but significant, even with a measure of working memory capacity included in the model. This may suggest that the relation between mind wandering and sleep disturbances is not due to reduced executive cognitive control. The association between sleep disturbances and the degree of unguided thoughts is arguably consistent with neural research of reduced functional connectivity in default mode network and hippocampal regions^43^, among other regions^44^, following sleep deprivation. Default mode network activity has previously been linked to task- and stimulus-independent thoughts^45^. It may be that sleep deprivation increases spontaneous thoughts because semantic networks have not been thoroughly consolidated, or, more generally, people may shift more easily into hypnagogic/hypnopompic moments, which may decrease overall cognitive stability^46^.

We did not test any hypotheses pertaining to a happy mood and our exploratory findings yielded no effects of happy mood induction on task-unrelated to unguided thoughts. To summarize, Seibert and Ellis^47^ found a rise in task-unrelated thoughts following happy mood induction compared to neutral mood induction, whereas we and Smallwood and colleagues^3^ did not. One discrepancy is that the former study asked participants to list all the thoughts they had during the task (free recall), whereas the other studies used forced choice prompts and questionnaires. A possibility is that the former method emphasized variety of contents, and this effect is arguably consistent with the broaden-and-build theory of positive emotions, which posits that a positive mood promotes exploration and widen the perspective^19^. In addition, a poor night of sleep is associated with a dysphoric mood, which could increase ruminations and decrease performance in cognitive tasks^48^. More research on the interactions among mood, sleep disturbances, and cognitive performance are clearly indicated.

It is important to acknowledge that we measured sleep disturbances prior to measures of task-unrelated thoughts and cognitive testing, so it is possible that participants were primed to think about sleep difficulties while performing the remainder of the study. Additionally, we cannot draw causal inferences from any observed association with sleep disturbances. Regarding the temporal association between mind wandering and sleep, a recent longitudinal study indicated that self-reported sleep disturbances on the night are likely to be followed by increased mind wandering the next day, but the study did not find a significant reversed association between mind wandering on the day and sleep disturbances the next night^49^. A second limitation of the present study is the use of self-reports for measuring sleep disturbances and mind wandering. Although other studies have shown that these measures correlate with the corresponding physiological and behavioural indices^2,50^, self-report biases could still account for some of the shared variance between these variables. A third limitation is that we did not distinguish between external distractions (e.g., being disturbed by someone talking on the phone) and stimulus-independent mind wandering (e.g., imagining the next vacation). We attempted to minimize the number of questions for the thought probes to prevent satisficing, and we instructed participants to complete the experiment alone and in quiet settings to reduce the likelihood of external distractions.

Considering that the sad mood induction in our study related to task-unrelated thoughts (small-to-medium effect size) and extended it to unguided thoughts (small effect size), researchers should exercise caution or avoid inducing a sad mood in circumstances in which unfocused thought may be risky, such as using heavy machinery or driving in traffic^51^). Although the sad mood induction was followed by weakly reduced efforts in concentrating on the task at hand, measuring emotion regulation strategies as a moderator could help elucidate the interactions between mood, cognitive abilities, and task-unrelated thoughts.

## Supplementary information

Dataset 1 & 2.

## Data Availability

The datasets analysed in the current study are available as supplementary files. These datasets include mean scale scores, but item-by-item or trial-by-trial scores are available from the corresponding author on reasonable request.
